# Transient activation of mTORC1 signaling in skeletal muscle is independent of Akt1 regulation

**DOI:** 10.14814/phy2.14599

**Published:** 2020-10-10

**Authors:** Mitsunori Miyazaki, Nobuki Moriya, Tohru Takemasa

**Affiliations:** ^1^ Department of Physical Therapy School of Rehabilitation Sciences Health Sciences University of Hokkaido Hokkaido Japan; ^2^ Department of Rehabilitation Faculty of Medical Science and Welfare Tohoku Bunka Gakuen University Miyagi Japan; ^3^ Graduate School of Comprehensive Human Sciences University of Tsukuba Ibaraki Japan

**Keywords:** Akt1, mechanistic target of rapamycin, protein synthesis, satellite cells

## Abstract

The regulation of cellular protein synthesis is a critical determinant of skeletal muscle growth and hypertrophy in response to an increased workload such as resistance exercise. The mechanistic target of rapamycin complex 1 (mTORC1) and its upstream protein kinase Akt1 have been implicated as a central signaling pathway that regulates protein synthesis in the skeletal muscle; however, the precise molecular regulation of mTORC1 activity is largely unknown. This study employed germline Akt1 knockout (KO) mice to examine whether upstream Akt1 regulation is necessary for the acute activation of mTORC1 signaling in the plantaris muscle following mechanical overload. The phosphorylation states of S6 kinase 1, ribosomal protein S6, and eukaryotic translation initiation factor 4E‐binding protein 1 which show the functional activity of mTORC1 signaling, were significantly increased in the skeletal muscle of both wildtype and Akt1 KO mice following an acute bout (3 and 12 hr) of mechanical overload. Akt1 deficiency did not affect load‐induced alteration of insulin‐like growth factor‐1 (IGF‐1)/IGF receptor mRNA expression. Also, no effect of Akt1 deficiency was observed on the overload‐induced increase in the gene expressions of pax7 and myogenic regulatory factor of myogenin. These observations show that the upstream IGF‐1/Akt1 regulation is dispensable for the acute activation of mTORC1 signaling and regulation of satellite cells in response to mechanical overload.

## Graphical abstract

In this study, the germline Akt1 knockout mice were used to examine whether upstream Akt1 regulation is necessary for the acute activation of mechanistic target of rapamycin complex 1 (mTORC1) signaling in the plantaris muscle following mechanical overload. We identified that upstream Akt1 regulation is dispensable for the acute activation of mTORC1 signaling.

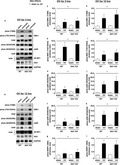

## 1 INTRODUCTION

The maintenance of skeletal muscle mass is crucial for human health and quality of life, because it is a critical determinant of locomotion and whole body's metabolisms that contribute to the reduced risk of morbidity/mortality under the diverse conditions such as disuse, aging, and several chronic diseases (Atherton et al., 2016; McLeod et al., 2016; Wolfe, 2006). The total mass of mature skeletal muscle is generally determined by the net dynamic balance between protein synthesis and degradation (Miyazaki & Esser, 1985). It has been indicated that the enhancement of protein synthesis in the skeletal muscle is primarily induced by the activation of mechanistic target of rapamycin complex 1 (mTORC1)‐dependent signaling (Goodman, 2014; Moriya & Miyazaki, 2018). The most well‐characterized upstream mechanism that regulates mTORC1 signaling in the skeletal muscle is through the growth factor (e.g., insulin/insulin‐like growth factor‐1 [IGF‐1])‐dependent activation of the phosphoinositide 3‐kinase (PI3K)/Akt pathway (Bodine et al., 2001; Miyazaki et al., 2010; Rommel et al., 2001). The ligand binding of insulin/IGF‐1 to the IGF receptor (IGFR) activates the receptor tyrosine kinase, which, then initiating a cascade reaction of downstream signaling, leads to the activation of the PI3K/Akt/mTORC1 pathway. The activation of mTORC1 signaling causes enhanced protein translational machinery through the phosphorylation of ribosomal protein s6 kinase (S6K1) and the translational repressor eukaryotic initiation factor 4E‐binding protein 1 (4E‐BP1) (Fingar et al., 2002; Laplante & Sabatini, 2012).

To date, many studies have demonstrated that the activation of PI3K/Akt/mTORC1 pathway in the skeletal muscle results in a robust increase in muscle cell size and total protein content in vitro and in vivo (Blaauw et al., 2009; Bodine et al., 2001; Goodman et al., 2011; Miyazaki et al., 2011; Rommel et al., 2001; Vyas et al., 2002). In addition, treatment with mTORC1 inhibitor rapamycin cancels the hypertrophic responses of skeletal muscle that are induced by the activation of PI3K/AKT/mTORC1 pathway (Bodine et al., 2001; Marabita et al., 2016; Rommel et al., 2001). These earlier reports clearly indicated that the inputs from upstream PI3K/Akt pathway is sufficient to induce skeletal muscle hypertrophy by the activation of mTORC1 signaling.

Despite this critical role of mTORC1 signaling in the regulation of muscle mass, the necessity of upstream IGF‐1/PI3K/Akt‐dependent regulation for the activation of mTORC1 and its subsequent muscle growth is still under discussion. Previously, we have reported that the activation of mTORC1 at a relatively earlier phase (within 24 hr) of mechanical overload in the skeletal muscle occurs independently of PI3K/Akt signaling (Miyazaki et al., 2011). Of the three different isoforms of Akt (Akt1, Akt2, and Akt3), Akt1 has been suggested to mediate cell growth mechanisms and regulate cell size (Chen et al., 2001; Cho et al., 2001; Wilson & Rotwein, 2007). Using the germline Akt1 knockout (KO) mice, we showed that Akt1 deficiency did not affect the load‐induced activation of mTORC1 signaling and the subsequent enhancement of protein synthesis in the skeletal muscle (Moriya & Miyazaki, 2018). These earlier reports from our lab and other findings (Hornberger et al., 2005; Hamilton et al., 2014; O'Neil et al., 2009; Witkowski et al., 2010) have suggested that the mechanical load‐induced activation of mTORC1 signaling and subsequent enhancement of protein synthesis in the skeletal muscle occurs independently of IGF‐1/PI3K/Akt1 regulation. However, the limitation of our earlier study was that all measurements including the activation status of mTORC1 signaling and cell biosynthesis events in the skeletal muscle of Akt1 KO mice were made during relatively later time points (~14 days following the onset of mechanical overload) and acute effects on mTORC1 activation were not investigated. Therefore, in this study, we examined whether regulatory inputs from upstream protein kinase Akt1‐dependent pathway are essential in the activation of mTORC1 signaling in the skeletal muscle following the acute onset of mechanical overload.

## 2 MATERIALS AND METHODS

### 2.1 Antibodies

phospho‐S6K1 (T389, Cat#: 9205), phospho‐S6K1 (T421/S424, Cat#: 9204), phos‐rpS6 (S235/236, Cat#: 4858), phos‐rpS6 (S240/244, Cat#: 5364), rpS6 (Cat#: 2317), 4E‐BP1 (Cat#: 9644), phos‐mTOR (S2448, Cat#: 5536), mTOR (Cat#: 2972), phos‐Akt (S473, Cat#: 4060), phos‐Akt (T308, Cat#: 5106), Akt (pan, Cat#: 4691), phos‐Akt1 (S473, Cat#: 9018), Akt1 (Cat#: 2967) were from Cell Signaling Technology. glyceraldehyde‐3‐phosphate dehydrogenase (GAPDH; Cat#: sc‐32233) and S6K1 (Cat#: sc‐230) were from Santa Cruz Biotechnology (Santa Cruz, CA, USA). IRDye 800CW Goat anti‐Mouse IgG (Cat#: 926‐32210) and IRDye 680LT Goat anti‐Rabbit IgG (Cat#: 926‐68021) were from LI‐COR Biosciences.

### 2.2 Animal care and use

All experimental procedures performed in this study were conducted in accordance with the institutional guidelines provided for the care and use of laboratory animals, which were approved by the Animal Ethics and Research Committee of the Health Sciences University of Hokkaido (no. 19‐060). Animals were housed in a temperature‐controlled and humidity‐controlled room and maintained on a 12‐hr light/12‐hr dark cycle with access to food and water ad libitum. We employed male C57BL/6JJmsSlc mice (Japan SLC) in this study. Breeding pairs (first generation) of Akt1 heterozygote mice (B6.129P2‐*Akt1^tm1Mbb^*/J) were also purchased from Jackson Laboratory. The second/third generation of heterozygote mice was bred for producing Akt1 KO mice. Wild‐type (WT) siblings from the same breeding colony were used as controls. All mice were male, aged between 8 and 9 weeks.

### 2.3 Synergist tenotomy surgery

We performed bilateral tenotomy surgery as described previously (Goldberg, 1967) under anesthesia after the inhalation of isoflurane (2.0% isoflurane in air). This in vivo model induces compensatory hypertrophic growth of the plantaris muscle through mechanical overload resulting from the surgical removal of the tendons from the synergist muscles (gastrocnemius and soleus). At the end of each experimental period, the mice were anesthetized, and the plantaris muscle was excised, weighed, quickly frozen in liquid nitrogen, and stored at −80°C. After the completion of experimental treatments, the mice were euthanized by cervical dislocation under anesthesia.

### 2.4 RNA isolation and RT‐PCR

Total RNA was prepared using the RNeasy Fibrous Tissue Mini Kit (Qiagen) according to the manufacturer's directions. The isolated RNA was quantified using spectrophotometry (*λ* = 260 nm). First‐strand cDNA synthesis from total RNA (500 ng of total RNA for 10 μl of reaction solution) was performed using the PrimeScript RT Reagent Kit, according to the manufacturer's instruction. SYBR Premix Ex Taq II, Premix Ex Taq (Probe qPCR), and TaKaRa Thermal Cycler Dice Real Time System TP850 (Takara Bio) were employed for PCR amplification. Predesigned primer/probe sets for REDD1 (Mm00512504_g1), REDD2 (Mm00513313_m1), and GAPDH (Mm99999915_g1) mRNAs were obtained from Thermo Fisher Scientific. Pax7 (Mm.PT.58.12398641), Mrf4 (Mm.PT.58.33344984), Myf5 (Mm.PT.58.5271235), Myod1 (Mm.PT.58.8193525), and Myogenin (Mm.PT.58.6732917) were obtained from Integrated DNA Technologies. Primer sequences (5ʹ–3ʹ, forward and reverse, respectively) for SYBR assay were designed as follows: Igf1, TACTTCAACAAGCCCACAGGC and ATAGAGCGGGCTGCTTTTGT (Kir et al., 2014); IGFR, GAGAATTTCCTTCACAATTCCATC and CACTTGCATGACGTCTCTCC (Li & Rana, 2012). Expression levels of each studied gene were determined by the 2^−ΔΔCT^ method while referencing glyceraldehyde‐3‐phosphate dehydrogenase (GAPDH) as an internal control. Absolute Ct values of GAPDH PCR amplification for each sample were stable (between 19 and 20 cycles) and no significant effects of genotype (WT vs. Akt1 KO) or experimental treatment (sham vs. mechanical overload) was observed.

### 2.5 Protein extraction and western blotting

Tissue samples were lysed in an ice‐cold RIPA buffer (1% NP‐40, 0.5% sodium deoxycholate, 0.1% SDS, 50 mM NaCl, 20 mM Tris–HCl (pH, 7.6), 1 mM PMSF, 5 mM benzamidine, 1 mM EDTA, 5 mM N‐ethylmaleimide, 50 mM NaF, 25 mM B‐glycerophosphate, 1 mM sodium orthovanadate, and 1× protease inhibitor cocktail [Nacalai Tesque, Kyoto, Japan]). The lysed samples were then centrifuged at 16,000× g for 10 min at 4°C, and the supernatants were collected for analysis. The protein concentration was determined using the BCA protein assay kit (Thermo Fisher Scientific). Protein samples were separated using a precast polyacrylamide gel system (e‐PAGEL; ATTO) and transferred to the PVDF membranes. Thereafter, the membranes were blocked in Odyssey Blocking Buffer and, then, incubated with the dilutions of each primary antibody. IRDye 800CW goat anti‐mouse IgG and IRDye 680LT goat anti‐rabbit IgG were used as secondary antibodies. Bound antibody complexes were scanned and quantified using the Odyssey Infrared Imaging System (LI‐COR Biosciences).

### 2.6 Statistical analysis

All results are reported as means ± *SE* values. Time course analyses were compared by one‐way ANOVA followed by Dunnett's post hoc test. Multi‐group comparisons (genotype vs. treatment) were performed by two‐way ANOVA followed by Tukey's post hoc test. For all comparisons, the level of statistical significance was set at *p* < .05.

## 3 RESULTS

### 3.1 The transient activation of mTORC1 signaling in the skeletal muscle is independent of Akt1 regulation

**FIGURE 1:**
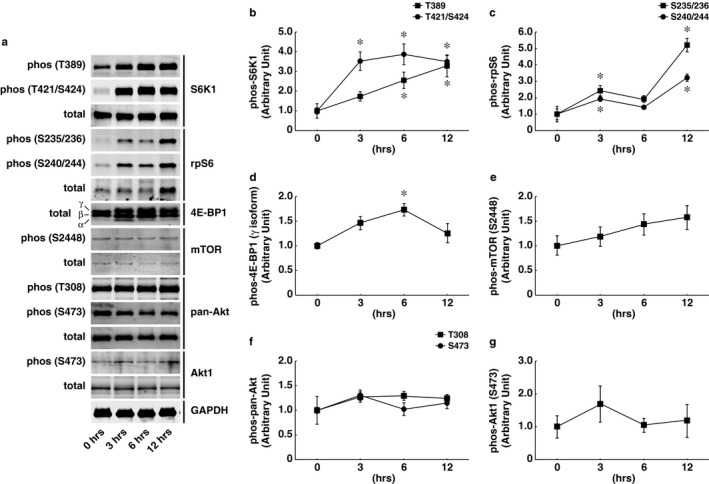
Temporal alteration of mechanistic target of rapamycin complex 1 (mTORC1) signaling in response to acute bout of mechanical overload. Mechanical overload‐induced phosphorylation of S6K1 (T389 and T421/S424 sites), rps6 (S235/236 and S240/244 sites), 4E‐BP1 (gamma isoform), mTOR (S2448 site), pan‐Akt (T308 and S473 sites), and Akt1 (S473) were determined in the plantaris muscle of male C57BL/6J mice. (a) Representative images showing phosphorylation status and total expression of each protein. GAPDH as an internal control. The relative (normalized to 0 hr) phosphorylation level of T389 and T421/S424 sites of S6K1 (b), S235/236 and S240/244 sites of rps6 (c), gamma isoform of 4E‐BP1 (d), S2448 site of mTOR (e), T308 and S473 sites of pan‐Akt (f), and S473 site of Akt1 (g) at each time point were quantified. *n* = 3/group. All results are expressed as mean ± *SE*. Significant differences: *compared to the control (0 hr) group, *p* < .05

Temporal alteration in the phosphorylation states of mTORC1‐dependent signaling in the plantaris muscle was determined after 3–12 hr of mechanical overload in C57BL/6J mice. As shown in Figure 1a–d, the phosphorylation states of S6K1, ribosomal protein S6 (rpS6), and 4E‐BP1 which were well‐characterized downstream targets of mTORC1 signaling, were significantly increased at 3–6 hr after mechanical overload as compared to the sham‐operated control group. Particularly, the phosphorylation of S6K1 at T389 and the gamma isoform of 4E‐BP1, that are often used as functional readouts of mTORC1 activity, were significantly increased at 6 hr following mechanical overload. S6K1 phosphorylation at the T421/S424 site (3.5‐fold) and rpS6 phosphorylation (2.4‐fold at the S235/236 and 1.9‐fold at the S240/244 sites) were also significantly increased at 3 hr, and remained significantly upregulated until 12 hr following mechanical overload. In contrast to the acute effect on mTORC1 activation, the phosphorylation of mTOR at S2448, a direct target site of Akt but not a determinant of intrinsic enzyme activity (Acosta‐Jaquez et al., 2009; Sekulic et al., 2000), was not altered at any time points until 12 hr after the initiation of mechanical overload (Figure 1e). The phosphorylation level of pan‐Akt at both the T308 and the S473 sites was also unchanged following the acute bout of mechanical overload (Figure 1f). Of the three Akt isoforms, Akt1 has been suggested to mediate cell growth machinery in skeletal muscle; therefore, we determined the effect of mechanical overload on Akt1 phosphorylation using Akt1‐specific phospho‐antibody. Similar to the phosphorylation kinetics of pan‐Akt, Akt1 phosphorylation was not significantly altered at any time points until 12 hr following mechanical overload (Figure 1g).

**FIGURE 2:**
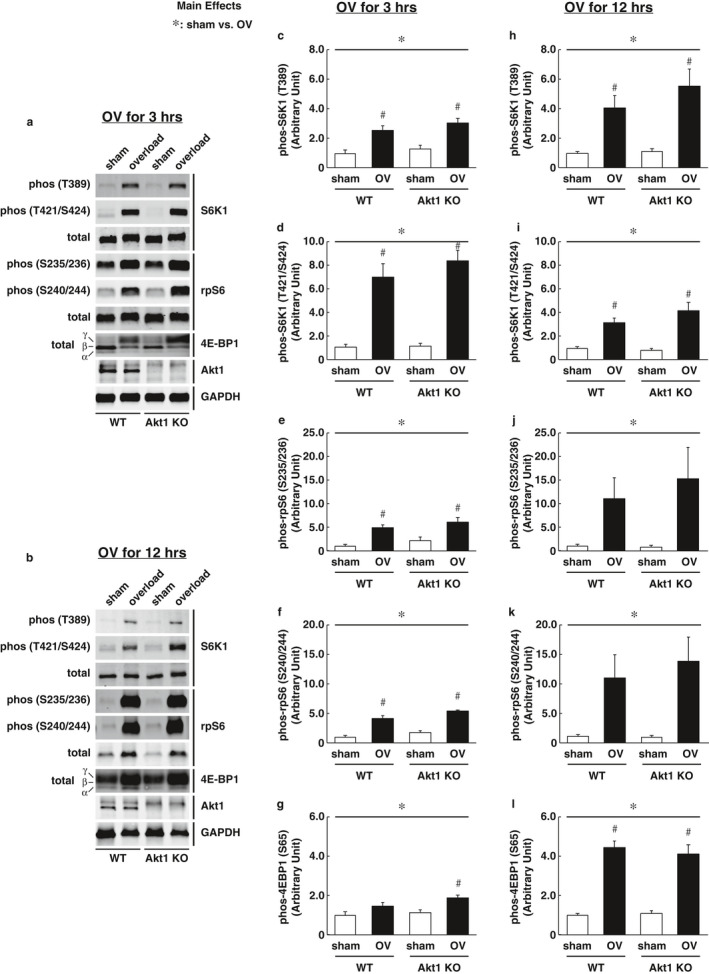
Akt1 deficiency did not affect the load‐induced activation of mechanistic target of rapamycin complex 1 signaling. Representative images of western blotting in 3 hr (a) and 12 hr (b) of mechanical overload. Phosphorylation states of S6K1, rps6, and 4E‐BP1 were determined and quantified (*n* = 6 in each group). (c, h) S6K1 at T389. (d, i) S6K1 at T421/S424. (e, j) rps6 at S235/236. (f, k) rps6 at S240/244. (g, l) 4E‐BP1 gamma isoform. Graphs of the (c)–(g) show the quantitative results of OV‐3 hr, and the (h)–(l) show the results of OV‐12‐hr. All results are expressed as mean ± *SE*. Significant differences: *, main effect of two‐way analysis of variance between experimental conditions (*p* < .05); #, between sham‐operated control and overload group for each genotype (*p* < .05)

**FIGURE 3:**
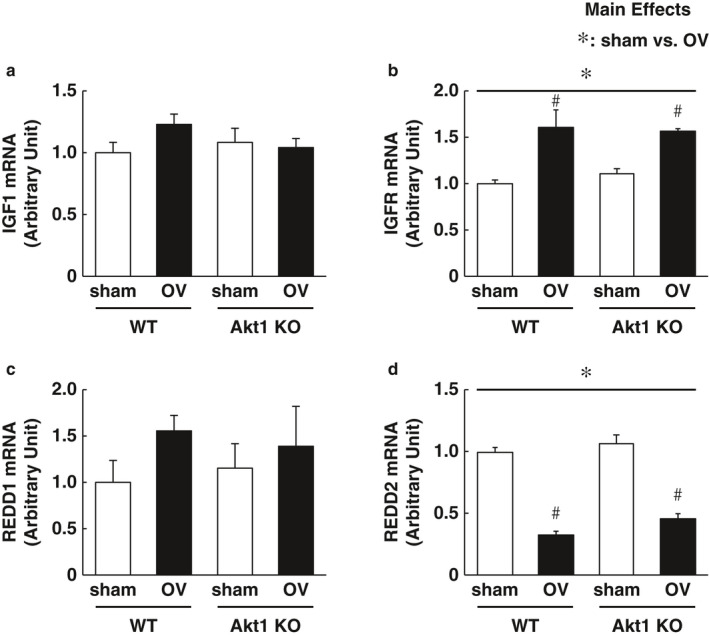
Load‐induced alteration of mRNAs expression in upstream regulator of mechanistic target of rapamycin complex 1 (mTORC1) signaling was independent of Akt1 regulation. Gene expressions of upstream regulator of mTORC1 signaling were determined (A–D). (a) insulin‐like growth factor‐1 (IGF‐1). (b) IGF receptor (IGFR). (c) Regulated in development and DNA damage response 1 (REDD1). (d) REDD2. *n* = 6 in each group. All results are expressed as mean ± *SE*. Significant differences: *, main effect of two‐way analysis of variance between experimental conditions (*p* < .05); #, between sham‐operated control and overload group for each genotype (*p* < .05)

Although phosphorylation status of Akt (both pan‐Akt and Akt1) was not significantly altered following the acute bout of mechanical overload, we cannot exclude the possibility that the endogenous phosphorylation of Akt1 in skeletal muscles is sufficient to mediate mTORC1 activation under overload conditions. Next, we used Akt1 KO mice to examine whether upstream Akt1 regulation is required for the acute activation of mTORC1 signaling in the plantaris muscle following mechanical overload. The phosphorylation states of S6K1 and rpS6 were significantly increased in both WT and Akt KO mice after 3 and 12 hr of mechanical overload. The load‐induced increase in 4E‐BP1 phosphorylation was rather higher in Akt1 KO mice than in the WT control mice following 3 hr of overload (Figure 2g). Importantly, no preventive effect of Akt1 deficiency was observed on the acute activation of mTORC1 signaling in the skeletal muscle at both 3 hr (Figure 2c–g) and 12 hr (Figure 2h–l) of mechanical overload. In addition, the expression levels of IGF‐1 mRNA were not altered by neither mechanical overload nor Akt1 deficiency. IGFR mRNA was significantly increased following 12 hr of mechanical overload in both WT and Akt KO mice, but Akt1 deficiency did not show any preventive effect on IGFR mRNA expression (Figure 3A,B). These observations clearly indicate that the acute activation of mTORC1 in response to mechanical overload occurs independently of upstream IGF‐1/PI3K/Akt1 regulation.

Having established that mTORC1 activation is occurred through a mechanism independently of IGF‐1/PI3K/Akt1, we next decided to investigate the Akt‐independent pathway that activates mTORC1 signaling. Regulated in development and DNA damage response 1 (REDD1) and its paralog, REDD2, have been reported to affect protein metabolism in skeletal muscle by acting as mTORC1 repressors through Akt‐dependent (Dennis et al., 2014) or independent manner (Miyazaki & Esser, 2009). Expression levels of REDD1 and REDD2 were also examined in this study. Although the gene expression of REDD1 was not altered, REDD2, an upstream negative regulator of mTORC1 signaling in the skeletal muscle was significantly downregulated following 12 hr of mechanical overload in both WT and Akt KO mice (Figure 3C,D).

### 3.2 Load‐induced activation of satellite cells and myogenic regulation were independent of Akt1 regulation

**FIGURE 4:**
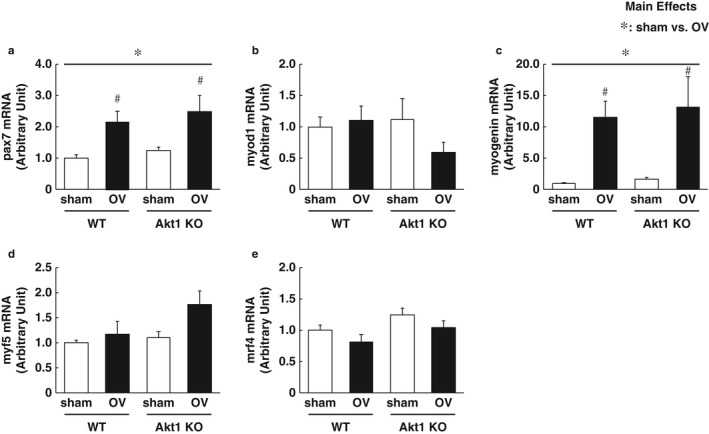
Load‐induced alteration of mRNAs expression in myogenic regulation was independent of Akt1 regulation. Gene expressions of pax7 and myogenic regulatory factors were determined (a–e). (a) pax7. (b) myod1. (c) myogenin. (d) myf5. (e) mrf4. *n* = 6 in each group. All results are expressed as mean ± *SE*. Significant differences: b*, main effect of two‐way analysis of variance between experimental conditions (*p* < .05); #, between sham‐operated control and overload group for each genotype (*p* < .05)

Previously, we have reported that Akt1 deficiency attenuates the hypertrophic response of skeletal muscle, which is likely through the blunted activation of satellite cell proliferation following 14 days of mechanical overload (Moriya & Miyazaki, 2018). To examine whether Akt1‐dependent regulation of satellite cells is still effective at the acute phase of mechanical overload in the skeletal muscle, we determined the transcripts expression of pax7 and myogenic regulatory factors. The gene expression of pax7, a typical marker of satellite cells, was significantly increased following 12 hr of mechanical overload in both WT and Akt KO mice (main effect of operation and Tukey's post hoc test between sham and overload, *p* < .05). The expression of myogenin mRNA was also significantly increased by acute mechanical overload in both WT and Akt1 KO mice. Mechanical overload did not affect the gene expressions of other myogenic regulatory factors, including myod1, myf5, and mrf4 (Figure 4. These data suggest that Akt1 deficiency did not affect the acute response of satellite cell activation and/or myogenic regulatory programs in response to mechanical overload.

## 4 DISCUSSION

The major question to address in this study was whether regulatory inputs from upstream Akt1‐dependent pathway are essential for the acute activation of mTORC1 signaling in response to an increased workload in the skeletal muscle. According to our results, the phosphorylation states of S6K1, rpS6, and 4E‐BP1 in the skeletal muscle following the acute bout of mechanical overload were occurred in both WT and Akt1 KO mice, thereby suggesting that mTORC1 activation was independent of upstream Akt1 regulation. Indeed, no alteration of IGF‐1 mRNA expression was observed in response to the mechanical overload of 12 hr in the skeletal muscle, whereas mTORC1‐dependent signaling was highly activated (Figures 2 and 3). These results are consistent with a previous study reporting that the overload‐induced expression of IGF‐1 mRNA in the rat's skeletal muscle was increased from 48 hr and at the later time point following the initiation of the workload (Adams et al., 1985).

Recent studies from several independent laboratories have supported the idea that muscle contraction‐ or mechanical load‐induced activation of mTORC1 signaling is not mediated by IGF‐1/PI3K/Akt pathway, especially during a relatively early stage of muscle hypertrophic response (Goodman et al., 2010; Hamilton et al., 2014; Ito et al., 2013; Maruyama et al., 1985; Miyazaki et al., 2011; Philp et al., 2011). These notions suggest the presence of potential mediators that convert acute mechanical stimulation into the activation of mTORC1 signaling in the skeletal muscle. Although it is still under intensive discussion, the potential key event that controls the activation status of mTORC1 occurs through the regulation of intracellular localization of mTORC1 to the lysosome and protein–protein interaction with its upstream regulators, tuberous sclerosis complex (TSC) and Ras homologue enriched in brain (Rheb; Betz & Hall, 2013). Previous reports have indicated that mitogen‐ or amino acid stimulation of nonmuscle cells resulted in the translocation of mTORC1 to the lysosome where it associates with Rheb, a direct stimulator of mTORC1 (Saito et al., 2005; Sancak et al., 2008). A farnesylated small G protein Rheb is anchored to the surface of the lysosome, which receives negative regulation by a GTPase‐activating protein TSC2. Since the inhibition of TSC2 GAP activity allows Rheb to accumulate in its active form, sequestered away of TSC2 from the lysosome leads to the enhanced mTORC1 activation. As a result, it promotes protein synthesis and cell growth (Carroll et al., 2016; Kim & Guan, 2019; Miyazaki et al., 2010). In the skeletal muscle, Jacobs and colleagues have reported that the acute bout of eccentric contractions in mouse tibialis anterior muscles enhanced the lysosomal translocation of mTOR, with the concomitant dissociation of TSC2 away from the lysosome to the cytosol, which likely contributed to mTORC1 activation (Jacobs et al., 2014). Song and coworkers have also confirmed that resistance exercise in the human skeletal muscle resulted in the rapid translocation of mTOR toward the lysosome, with the concurrent dissociation of TSC2 from Rheb, which would facilitate the interaction of mTOR and GTP‐Rheb (Song et al., 2017). Collectively, these studies suggested that the cellular trafficking of mTOR protein is a fundamentally important event to initiate protein synthesis and cell growth machinery during an early phase of muscle hypertrophic response.

While intracellular distribution of mTORC1 was not evaluated in this study, we confirmed the downregulation of REDD2 mRNA expression in response to the acute bout of mechanical overload. It has been indicated that a muscle‐specific stress‐responsive gene *REDD2* potently inhibits mTORC1 signaling by controlling the TSC2 function (Miyazaki & Esser, 2009). Previous studies conducted on human skeletal muscle have also reported that *REDD2* and its homolog *REDD1* plays a vital role in regulating mTORC1 activity and protein synthesis following an acute bout of resistance exercise (Drummond et al., 2008, 2009). Although the protein expression of REDD2 was not determined in this study due to the no availability of reliable antibody on a commercial basis, a very short cellular half‐life (less than 5 min) of REDD1/2 proteins (Katiyar et al., 2009; Kimball et al., 2008) allowed us to speculate that the alteration of REDD2 mRNA expression is likely reflective of mTORC1 activity. According to these observations, we suggest that an acute bout of mechanical loading in skeletal muscle may dampen the inhibition of REDD2 to mTORC1 signaling, likely by controlling the TSC2 function.

In contrast to previous data showing the diminished satellite cell proliferation in Akt1 KO mice following 2 weeks of mechanical overload (Moriya & Miyazaki, 2018), Akt1 deficiency showed no effect on the gene expressions of pax7 and myogenic regulatory factors in response to the acute bout of mechanical overload. Indeed, a previous in vitro study suggested that the expression levels and activity of Akt are essential for the development of normal myotube during the later stages of muscle differentiation (Gardner et al., 2012). These observations have suggested that Akt1‐dependent regulation is dispensable for the acute activation of satellite cells and may play a different role for controlling satellite cells during a relatively later phase following increased mechanical load on the skeletal muscle. Future studies should necessarily consider that Akt1‐dependent regulation would participate in other steps of satellite cell regulation, including commitment, differentiation, and fusion processes into the existing myofibers in response to the increased workload. As a note, since we used whole muscle tissue to prepare RNA samples, it is not possible to determine in this study whether mature skeletal muscle fibers or surrounding muscle satellite cells express the other myogenic regulators, except for Pax7. Another limitation was the use of germline Akt1 KO mice, which makes it impossible to determine whether the effect of the Akt1 deficiency is caused by mature skeletal muscle fibers or muscle satellite cells. Hence, it will be important in future studies to evaluate whether a specific deletion of Akt1 in either mature skeletal muscle or muscle satellite cells using the conditional KO model can affect the load‐induced activation of mTORC1 signaling and skeletal muscle hypertrophy.

## 5 CONCLUSION

We employed germline Akt1 KO mice to examine whether upstream Akt1 regulation is essential for the acute activation of mTORC1 signaling in the plantaris muscle following mechanical overload. The phosphorylation states of S6K1, rpS6, and 4E‐BP1 were increased in the skeletal muscle of both wildtype and Akt1 KO mice following an acute bout (3 and 12 hr) of mechanical overload. Also, no effect of Akt1 deficiency was observed on the overload‐induced alteration of mTORC1 upstream regulators (IGFR and REDD2) and myogenic regulatory factors (pax7 and myogenin). These observations demonstrate that the upstream IGF‐1/Akt1 regulation is dispensable for the acute activation of mTORC1 signaling and satellite cells regulation in response to mechanical overload.

## CONFLICT OF INTEREST

We have no conflict of interest to declare.

## AUTHOR CONTRIBUTION

M.M., N.M., and T.T. conceived and designed the project. M.M. and N.M. acquired, analyzed, and interpreted the data. M.M. wrote, revised, and edited the paper.

## ETHICAL STATEMENT

All experimental procedures performed in this study were conducted in accordance with the institutional guidelines provided for the care and use of laboratory animals, which were approved by the Animal Ethics and Research Committee of the Health Sciences University of Hokkaido (no. 19‐060).

## Data Availability

All relevant data are within the manuscript.
